# From E-Patients to AI Patients: The Tidal Wave Empowering Patients, Redefining Clinical Relationships, and Transforming Care

**DOI:** 10.2196/75794

**Published:** 2025-05-16

**Authors:** Susan S Woods, Sarah M Greene, Laura Adams, Grace Cordovano, Matthew F Hudson

**Affiliations:** 1 Fork Food Lab, 95 Darling Ave, South Portland, ME, 04106, United States; 2 National Academy of Medicine, Washington, DC, United States; 3 Enlighteningresults, LLC, New York, NY, United States; 4 Prisma Health, Greenville, SC, United States

**Keywords:** artificial intelligence, AI, large language models, LLM, participatory medicine, co-design, coproduction, internet, patients, caregivers, patient engagement, patient empowerment, digital health

## Abstract

Artificial intelligence (AI) and large language models offer significant potential to enhance many aspects of daily life. Patients and caregivers are increasingly using AI for their own knowledge and to address personal challenges. The growth of AI has been extraordinary; however, the field is only beginning to explore its intersection with participatory medicine. For many years, the *Journal of Participatory Medicine* has published insights on tech-enabled patient empowerment and strategies to enhance patient-clinician relationships. This theme issue, Patient and Consumer Use of AI for Health, will explore the use of AI for health from the perspective of patients and the public.

## Introduction

Artificial intelligence (AI) and large language models (LLMs) offer boundless potential to enhance many aspects of daily life. The promise of AI for health is profound: to discover new treatments, gain efficiencies, and deliver precision medicine—the right intervention to the right person at the right time [1]. Experts are effusive about AI, which can reduce cognitive workload, enhance prevention, and lower costs. Many blunt this enthusiasm with caution, as the field struggles to genuinely address AI ethics, accountability, privacy, and governance [2].

Along with the hope (and hype) of AI within health care, the public is swiftly taking AI into their own hands. Consumers are at the forefront in this era of AI. A survey conducted in January 2025 by Imagining the Digital Future Center found that 52% of US adults used ChatGPT, Gemini, CoPilot, or other LLMs. Among LLM users, half reported personal learning as their goal, and 39% sought information about physical or mental health [3]. Patients burdened with life-changing or rare conditions commonly search for the resources that they need to solve problems. As consumer costs of care keep rising and health care is relentlessly hard to navigate, patients and caregivers are gaining skills and intelligence using LLMs across a breadth of topics. These information seekers go beyond clinical content, using AI for personalized advice to tackle legal, financial, social, and many of life’s challenges.

While people may not realize the ubiquity of AI, millions interact with AI daily using assistants such as Siri or Alexa and streaming platforms such as Netflix and Spotify [4]. Launched in November 2022, ChatGPT reached 100 million users in 2 months and hundreds of millions of users by March 2024 [5]. This scorching adoption has been faster than for personal computers and the internet. In 2024, a total of 39.4% of US adults aged 18-64 years reported using generative AI, and 32% used it weekly. In contrast, 20% of the public used the internet 2 years after its launch, and 20% owned a computer after 3 years of availability. While price and ease of use play a role in the difference, the advancement of AI is without historic parallel.

Projections of the health AI market over the next decade are staggering, with estimates of US $27 billion in 2024 climbing to US $613 billion by 2034 [6]. At this early stage, the direct-to-consumer market may mature faster and more readily than inside health care [7]. Yet, current research on AI for health largely focuses on clinician and professional users. It is essential to study how AI can best serve patients while mitigating risks. Although papers on the use of AI by patients and the public are starting to emerge, we believe this is the first theme issue in a medical journal that is dedicated to the topic.

## Rise in AI in Health Care Delivery Settings

Across health care, AI tools vary in their capabilities and stage of adoption (eg, to analyze data or optimize workflows) [8]. LLMs currently evaluate x-rays and images and enhance radiologists’ diagnostic accuracy. AI is even in operating rooms, helping surgeons with the use of robotics during procedures. AI-enabled wearable devices gather patient data remotely to inform and augment cardiologists’ decision-making. AI is synthesizing vast volumes of data locked in electronic health records, transforming raw data into actionable information. AI is accelerating pharmaceutical development, expediting drug discovery, and reducing the costs of clinical trials [9]. Notably, patient-physician-scientist partnerships are expanding, and using AI for “drug repurposing,” or searching existing medications that work for rare diseases, is also accelerating [10].

For patients, the visibility of AI in health care is low but rising. AI scribes are being used to record human conversations during encounters and summarize visits. Automating the documentation of visits may realize a “holy grail” by giving clinicians more time for patients and families. One study found that a year after deploying AI scribes, most physicians had a positive experience. All patients in the study reported that AI had either a positive or neutral impact on the quality of their visit; only 8% of patients felt some level of discomfort [11]. These AI agents remain a work in progress, as AI documentation continues to gain accuracy and completeness.

Health systems are using AI-derived content to respond to patients’ emails. Research on AI automated responses suggests that patients find messages to be satisfactory, with many comparable to emails from physicians; moreover, patients rated some responses as more empathetic than human clinician replies [12]. While AI messaging may help, health systems recognize the inherent risks in responding with inaccurate or potentially harmful information. Further, ethical concerns have been raised when patients believe responses are from a human and not a computer, or if they cannot ascertain whether replies are written by AI [13].

AI will remodel the patient experience and affect patient-clinician relationships. AI assistants do not replace the need for human judgment, particularly in cases requiring nuanced decisions. Importantly, patient and public involvement in AI development and refinement are critical to improve value, ensure safety, and engender trust. Further, more attention is warranted on the growth of AI tools that patients and caregivers are using independently for their health [5].

## The (R)evolution of Patient and Public Agency and Empowerment

The 21st century will be the age of the net empowered medical end user, and the patient-driven online support networks of today will evolve into more robust and capable medical guidance systems that will allow end users to direct and control an ever-growing portion of their own medical care.

[Tom Ferguson, MD, 2002] [14]

Ferguson was a family physician and pioneer who advocated for consumer use of the internet, believing that clinicians had much to learn from patients and families. He observed that patients who possessed internet-derived knowledge were more involved in their health and their care—the hallmark of participatory medicine [15]. He presciently wrote about tech-savvy patients who disengage from doctors who do not support patients accessing online information for self-care.

Participatory medicine continues to evolve, albeit sluggishly. For over three decades, the internet has served patients as a powerful tool to access previously unavailable information and connect with peers [16]. This shift in how people manage their health also altered power dynamics at medical visits and led to the term “Dr. Google” [17]. While greater patient control and contribution unfolded, not all clinicians have been comfortable with patients online or serving in a new role as “guide” or “partner” rather than expert authority.

The *Journal of Participatory Medicine* (*JoPM*) has been a pioneer, contributing insights on tech-enabled patient empowerment and enhancing patient-clinician relationships. *JoPM*’s early content was published on the Society of Participatory Medicine website, edited by Charlie Smith, Joe Graedon, and Terry Graedon, from 2009 to 2017. Authors included luminaries such as Esther Dyson, George Lundberg, Jessie Gruman, Kurt Stange, Kate Lorig, “e-patient Dave” DeBronkart, and many others. In 2017, *JoPM* joined JMIR Publications as a peer-reviewed, open access journal to advance the science of participatory care (also referred to as coproduction and co-design). Published papers mirror the 15-year shift in relationships between patients, their health information, and their providers.

Health professionals often overestimate the risks of e-patients (patients and caregivers online) and underestimate their value [18]. Despite the long-standing evidence that a participatory decision-making style leads to greater patient satisfaction and trust in health professionals [19], medical educators and practitioners have yet to fully acknowledge that patients are already active managers of their care, failing to support patients in this role [20]. Yet the evidence is there: e-patients are more prepared, feel more in control of their care, and achieve better outcomes [21].

The value of patient-facing technology continues to soar. Patients can now access all their clinical notes and test results online, mandated by the 21st Century Cures Act. Opening notes ushered in a wealth of research showing benefits of shared data to patients and families [22]. Along with technology empowering patients, health care has adopted a more holistic perspective. This shifted patient inquiry from “What is the matter with you?” to “What matters to you?” This approach robustly assesses social drivers of health and clarifies patient context, allowing care teams to codevelop realistic and achievable care plans.

The democratization of information and near-universal access to the internet have help innumerable patients. Not all health care organizations celebrate such progress, however. Patient portals, a splendid tool for patients, also contribute to clinician’s administrative burden. Patient messaging volume has escalated, leading some organizations to charge for e-communication. Real-time access to laboratory, imaging, and pathology tests causes apprehension among clinicians who feel unprepared when patients are first to see results. Some clinicians also believe that patient access to their health information threatens therapeutic relationships and extends the length of visits [23].

AI advancements introduce a range of new challenges. Too much information may overwhelm patients and caregivers and add uncertainty and anxiety when seeking credible and reliable resources, while a lack of information can cause patient anxiety. Lack of internet connectivity or device access excludes patients from benefiting from digital tools [24]. Consequently, there are expectations that AI tools—somewhat paradoxically—will solve the problem of too much information and narrow the digital divide. Then again, AI-derived outputs are knowingly biased since public access to peer-reviewed research is often behind “paywalls” that are restricted to institutional subscribers.

## AI Patients and Consumers: It Is Already Here

Often considered “the future,” AI is here today and integrated into everyday life. Positioned to facilitate moving patients and families into this new age, AI amplifies earlier e-patient behavior to obtain relevant health information, increase patient control over health and care, enhance health literacy, stimulate coequal contributions in decision-making processes, and enhance relationships with clinicians. Society has moved from e-patients to AI patients.

The public use of AI will grow exponentially. AI assistants will be increasingly used to explore symptoms; help with managing chronic diseases; and offer advice on nutrition, exercise, and more. AI-enabled wearable and smart devices, now used for people to track their activities to make real-time adjustments, will flourish. Those with life-altering diagnoses or rare diseases will use AI as a research assistant and copilot to obtain tailored data to guide treatment planning, especially when traditional forms of care have been exhausted. AI-powered peer support will transform into patient-led knowledge networks, and caregivers will use AI tools to monitor their loved ones while aiming to lower their stress.

As AI augments traditional care, there will be consequences. One example is the surge of low-cost AI chatbots targeting adolescents and young adults to address mood and mental health. Promoted as “personal intelligence” tools, these on-demand chatbots engage users to reflect on their feelings, organize thoughts, and help make decisions. Early research on AI chatbots for anxiety and depression has been mixed. Some studies show reductions in symptoms and perceived loneliness among frequent users [25]. Challenges, however, include emotional attachment and user dependency, lack of professional oversight, harmful messaging, and legal and privacy issues [26].

As health systems use “virtual first” approaches to care, boundaries between patients using AI alone versus AI with clinicians may become blurred. AI accuracy and trustworthiness will require incorporating human intelligence and feedback (human in the loop) to improve its accuracy and earn trust. Still, because patients’ needs are often not being met, any tools that can help patients navigate care and solve problems could be valuable.

## The Need for Research, Education, and Co-Design

These challenges underscore the need for research to identify both AI benefits and risks, especially among vulnerable populations. Like the e-patient era, the AI patient era may underestimate the significance of people using information to manage their health. Unlike the past, however—where risks to patients online were overestimated—AI stakeholders may underestimate the risks of AI to patients. These tools are powerful yet presently subject to only minimal regulation and governance. AI researchers must study how patients and caregivers use AI and assess how it impacts their lives. AI developments need to be co-designed with patients and ensure that governance includes rigorous regulatory and other guardrails, thereby preventing harm while promoting beneficial use [27]. Reputable organizations provide salient approaches to meaningfully involve patients and the public in research and care delivery, including the Patient-Centered Outcomes Research Institute [28] and the UK Standards for Public Involvement [29]. Critical guidelines are available from the National Academy of Medicine’s AI Code of Conduct [30] and The Light Collective’s AI Rights for Patients, which outlines seven patient rights critical to the development and deployment of AI in health care [31].

Finally, there is a fundamental educational imperative to equip patients and consumers with the knowledge and skills necessary to critically engage with AI tools for health. Educational offerings should encompass basic concepts and principles of AI and LLMs, effective prompting strategies, and understanding that machine learning systems may generate inaccurate or misleading outputs (ie, “hallucinations”). Learners must be aware of AI’s considerable variability in quality, transparency, equity, and reliability. Such instruction is essential to ensure individuals use AI tools responsibly and effectively to support their health and well-being.

Our journal’s theme issue, Patient and Consumer Use of AI for Health, begins exploring the use of AI for health from the perspective of patients and the public. The scope of our special issue posits the following:

- What is the patient and caregiver experience using AI tools for health and care?
- How can patients, caregivers, and the public use AI for maximum benefit?
- What are the risks and unintended consequences of AI use by patients, and how can these be mitigated?
- What is the impact of AI derived from health systems and presented to patients?
- How does AI affect patient-clinician relationships or patient–health care relationships?
- How can patient and public involvement be a standard in designing, developing, and deploying AI for health?

The growth of AI has been extraordinary; however, the field is only beginning to explore its intersection with participatory medicine. Health care must expand its “patient-centered” views and embrace the power that AI use affords patients and caregivers, as they are not seeking permission but are already using LLMs. Researchers must investigate consumer use of AI, co-designing studies with patients and caregivers, and determine how to avoid unintended consequences. The innovation community must embrace patient and public involvement throughout the development life cycle. We hope that this work inspires others to contribute to this new era of #PatientsUseAI.

## Abbreviations

AI: artificial intelligence
*JoPM*: *Journal of Participatory Medicine*
LLM: large language model
